# Screen-detected *vs* symptomatic breast cancer: is improved survival due to stage migration alone?

**DOI:** 10.1038/sj.bjc.6604368

**Published:** 2008-05-27

**Authors:** G C Wishart, D C Greenberg, P D Britton, P Chou, C H Brown, A D Purushotham, S W Duffy

**Affiliations:** 1 Cambridge Breast Unit, Box 97, Addenbrooke's Hospital, Hills Road, Cambridge CB2 2QQ, UK; 2 Eastern Cancer Registration and Information Centre (ECRIC) Unit C, Magog Court, Shelford Bottom, Hinton Way, Cambridge CB22 3AD, UK; 3 Cancer Research UK Centre for Epidemiology, Mathematics and Statistics, Wolfson Institute of Preventive Medicine, Charterhouse Square, London EC1M 6BQ, UK; 4 King's College London, Department of Academic Oncology, Guy's Hospital, Guy's and St Thomas’ NHS Foundation Trust, 3rd Floor, Thomas Guy House, St Thomas Street, London SE1 9RT, UK

**Keywords:** breast cancer, breast screening, survival

## Abstract

This paper examines whether screen-detected breast cancer confers additional prognostic benefit to the patient, over and above that expected by any shift in stage at presentation. In all, 5604 women (aged 50–70 years) diagnosed with invasive breast cancer between 1998 and 2003 were identified by the Eastern Cancer Registration and Information Centre (ECRIC) and mammographic screening status was determined. Using proportional hazards regression, we estimated the effect of screen detection compared with symptomatic diagnosis on 5-year survival unadjusted, then adjusted for age and Nottingham Prognostic Index (NPI). A total of 72% of the survival benefit associated with screen-detected breast cancer can be accounted for by age and shift in NPI. Survival analysis by continuous NPI showed a small but systematic survival benefit for screen-detected cancers at each NPI value. These data show that although most of the screen-detected survival advantage is due to a shift in NPI, the mode of detection does impact on survival in patients with equivalent NPI scores. This residual survival benefit is small but significant, and is likely to be due to differences in tumour biology. Current prognostication tools may, therefore, overestimate the benefit of systemic treatments in screen-detected cancers and lead to overtreatment of these patients.

Breast cancer remains a major UK public health issue with over 40 000 newly diagnosed patients and 15 000 deaths per annum. Following publication of initial trials that showed that mammographic screening could reduce breast cancer mortality (Shapiro *et al*, 1971; Tabar *et al*, 1985), the National Health Service Breast Screening Programme (NHSBSP) was introduced in 1988 in the United Kingdom and offered 3 yearly mammographic screening initially to women aged 50–65 years. The upper age limit is now 69 years and from 2008 screening onwards it will be offered to women aged 47–73 years. A recent overview of published screening trials has confirmed a reduction in breast cancer mortality of 21% in woman attending for mammographic screening (Nyström *et al*, 2002). The rational for this survival benefit is that screening enables breast cancers to be diagnosed at an earlier stage of disease. It is now well documented that screen-detected cancers are generally smaller, of lower grade and less likely to have axillary lymph node involvement (Weaver *et al*, 2006).

The Nottingham Prognostic Index (NPI) (Todd *et al*, 1987), a prognostic tool based on tumour size, grade and lymph node status, allocates individual patients to one out of five prognostic groups with quite different survival predictions. For many years, it has been assumed that the survival benefit associated with screen-detected cancers is due to stage shift, with these cancers presenting in a better prognostic group and intuitively one would expect that cancers, which have an equivalent NPI, would have the same prognosis regardless of their mode (i.e., screening or symptomatic) of detection. Two recent papers, however, have suggested that screen detection confers an additional survival benefit beyond stage shift (Shen *et al*, 2005) and reduces the risk of systemic recurrence when compared with symptomatic cancers of a similar stage (Joensuu *et al*, 2004). This current paper, therefore, aims to examine whether a cancer detected by mammographic screening confers additional prognostic benefit to the patient over and above that expected by the improved NPI stage shift.

## MATERIALS AND METHODS

Female patients diagnosed between 1998 and 2003 with invasive breast cancer (ICD10 site code C50^*^), and aged between 50 and 70 years at diagnosis, were identified by the Eastern Cancer Registration and Information Centre (ECRIC). During this period, ECRIC covered a population of approximately 2.75 million people in the counties of Bedfordshire, Cambridgeshire, Norfolk and Suffolk. Mammographic screening status of these cancers was determined by matching data from the 11 breast screening units in the East of England with the ECRIC registry database. These screening unit data were received by ECRIC via the East of England Breast Screening Quality Assurance Reference Centre.

Mode of detection has been classified as either screen detected (including both prevalent and incident cases) or symptomatic (regardless of whether the patients had ever been screened). We identified 5604 female patients with breast cancer, with over 97% confirmed histologically, and determined the current vital status of these patients. The vital status of each individual patient in this study was followed up actively by ECRIC in early 2007 by querying the National Health Service Strategic Tracing Service. Thus, it is expected that these data are substantially complete and reliable. Data elements recorded by ECRIC include age at diagnosis, pathological tumour size, number of nodes excised and status, treatment type (wide local excision, mastectomy, axillary surgery, radiotherapy, chemotherapy and hormone therapy) and index of multiple deprivation based on patient's electoral ward of residence. The primary sources of registration and treatment data are reports from all pathology laboratories and hospital patient notes, which are viewed by registry staff who are either based at all major NHS hospitals in the region or visit them on at least a monthly basis. Both electronic and paper-based reports are received by the registry, so a high level of completeness of registration is also expected.

From the tumour size, lymph node status and histological grade, we calculated the NPI for each case. The NPI has been validated in other breast cancer populations and allows assessment of the effect of different treatments in each of the five different prognostic groups, excellent (NPI<2.4), very good (2.4<NPI<3.4), moderate 1 (3.4<NPI<4.4), moderate 2 (4.4<NPI<5.4) and poor (NPI⩾5.4) (Sundquist *et al*, 1999). We estimated the effect of continuous NPI in screen-detected and symptomatic disease, and derived corresponding fitted 5-year survival curves.

Differences between screen-detected and symptomatic patients with respect to categorical variables were assessed using the *χ*^2^ test. For analysis of the effect of screen detection, we analysed data only from subjects aged 50–69 years at diagnosis, the age range of the NHSBSP. Survival was analysed using proportional hazards regression (Cox, 1972). We first estimated the effect of screen detection as compared with symptomatic diagnosis on survival unadjusted, then adjusted for age and NPI. The method of Freedman *et al* (1992) was used to estimate the percentage of the effect of screen detection on survival that can be attributed to other factors such as NPI.

## RESULTS

Table 1 shows the frequencies by age and NPI for screen-detected and symptomatic patients. Screen-detected patients were significantly younger (*P*<0.001) and were more likely to have favourable NPI categories (*P*<0.001) than the symptomatic patients. Screen-detected patients were also less likely to have NPI unknown.

**Figure 1:**
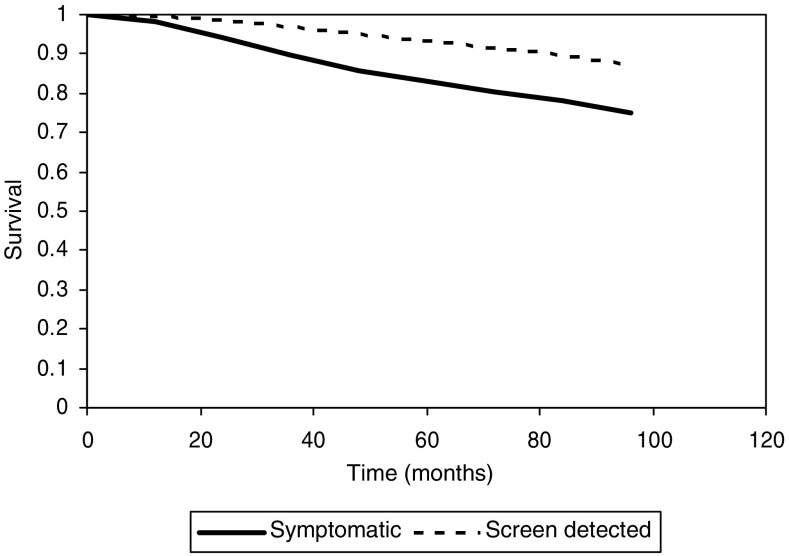
Survival by detection mode.

Figure 1 shows survival by time for screen-detected and symptomatic patients. Table 2 shows the results of Cox's regression analysis from the univariate models for the separate effects of each of NPI, age and detection on survival, and the multivariate model with each factor adjusted for the two others. Better survival was observed in younger patients, especially those with favourable NPI and screen-detected patients. All three factors had highly significant effects on survival in the univariate analyses (*P*<0.001 in all cases).

In the multivariate model, all three factors retained their statistical significance, but the effect of screen detection on survival was much attenuated after adjustment for age and NPI, with the relative hazard changing from 0.43 to 0.79. Freedman's estimate of the proportion of the effect of screen detection on survival accounted for by age and NPI was 72%.

Although there is some evidence that histological grade may deteriorate as the tumour progresses and that early detection can arrest this (Duffy *et al*, 1991), it is also at least partly an innate biological feature. We, therefore, also estimated the effect of adjustment for size and node status only. The adjusted relative hazards for screen detection and the Freedman estimate of the proportion of the screening effect accounted for by adjustment for various factors are shown in Table 3. Adjustment for size and node status takes account of 49% of the effect of screen detection on survival, shifting the relative hazard from 0.43 to 0.66. Adjustment for NPI (the addition of histological grade to size and node status) accounts for 67% of the screen detection effect, shifting the relative hazard to 0.76. Adjustment for both NPI and age accounts for 72% of the effect and moves the relative hazard to 0.79.

**Figure 2:**
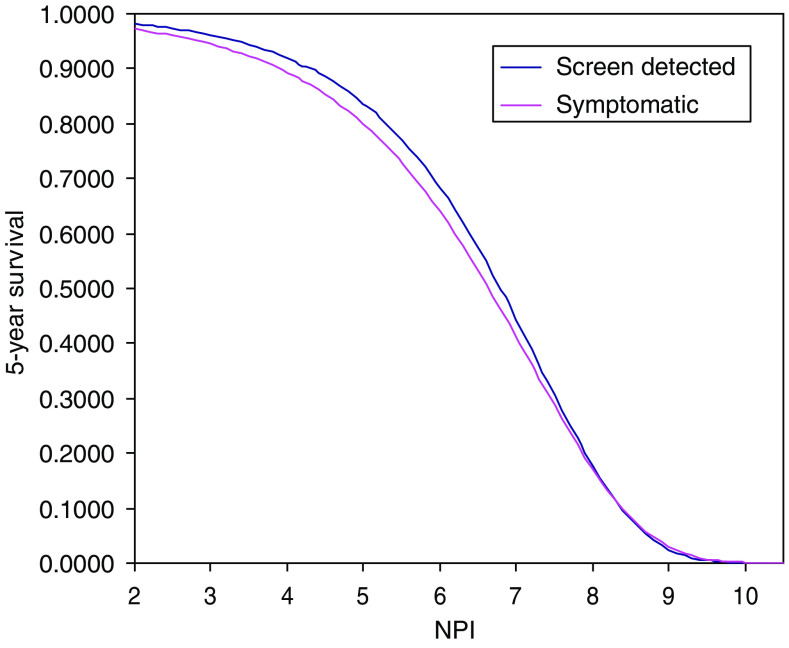
Fitted 5-year survival by continuous NPI (*P*=0.01).

The 5-year overall survival figures for all patients, and by mode of detection, are shown in Table 4. The greatest absolute survival benefit for screen-detected cancers is seen in the bottom two prognostic groups with a 10% absolute difference in the moderate 2 group. Survival analysis by continuous NPI showed a small but systematic survival benefit for screen-detected cancers at each NPI value (Figure 2).

## DISCUSSION

We analysed 5-year survival data of women aged 50–70 years diagnosed with invasive breast cancer in the East of England. Our results confirm a strong survival advantage of screening compared with symptomatic detection. They show that the majority of this effect can be attributed to a shift in NPI. This is best illustrated in Figure 2, where there is a small survival benefit for screen detection at each NPI value. After adjustment for NPI and age, only 28% of the screen detection survival advantage remained to be explained. Some of this is likely to be due to residual lead-time and length bias. In lead-time bias, screening advances the time of diagnosis so there is an artificial increase in survival time from diagnosis whatever the effect (or lack of effect) on the ultimate time of death. Length bias is the phenomenon whereby slower growing cancers remain in the preclinical detectable phase longer than faster growing cancers, and therefore screening will inevitably detect proportionally more slower growing, better prognosis cancers than those seen in the symptomatic population. Although it is likely that most of both biases should have been accounted for by NPI shift, quantification of this is the subject of ongoing research.

The remainder of the survival advantage is likely to be due to additional biological differences between screen-detected and symptomatic cancers including rates of hormone receptor positivity, HER 2 status and other biological factors. A recent tissue microarray study examined expression of a panel of 13 biomarkers (including ER, PR and HER2), in two independent case series and found that only Bcl-2 retained prognostic significance independent of NPI on multivariate analysis (Callagy *et al*, 2006). It is possible, however, that a number of biological markers, that on their own are not significant, contribute to the remaining 28% survival advantage seen with screen-detected cancers and this is the subject of ongoing research. The interesting point is that the majority of the survival effect of screen detection is accounted for by a shift in NPI, with only 28% attributable to other factors. These results would appear to confirm previous studies that suggested that screen detection was an independent prognostic factor for both disease-specific survival (Shen *et al*, 2005) and distant recurrence (Joensuu *et al*, 2004).

Accurate prognostication plays an essential role in the selection of appropriate adjuvant therapy. At present, mode of detection is not taken into account when calculating the risk of recurrence or death or subsequent treatment benefits. If the survival data for all patients is used to calculate absolute treatment benefits for patients with screen-detected cancers, then it is possible that the potential benefits will be overestimated (Table 4). This, in turn, may lead to potential overtreatment of patients with screen-detected cancers. The authors recognise that the number of patients with NPI unknown is greater in the symptomatic group (34%) than the screen-detected group (17%), but this is unlikely to significantly impact on these findings.

These data confirm the known survival advantage for patients with screen-detected cancers. They show that although most of this advantage is due to a shift in NPI, the mode of detection does impact on survival in patients with equivalent NPI scores. This residual survival benefit is small but significant, and is likely to be due to differences in tumour biology between screen-detected and symptomatic cancers. Current prognostication tools that do not include known biological markers may overestimate the benefit of systemic treatments in screen-detected cancers and lead to overtreatment of these patients. A prognostic tool combining clinical, pathological and biological factors might allow more accurate prognostication, and more appropriate systemic therapy, for all patients with breast cancer regardless of their mode of detection.

## Figures and Tables

**Table 1:** Age and NPI category frequencies by detection mode

		**Number of patients (%)**	
**Factor**	**Category**	**Symptomatic**	**Screen detected**
Age (years)	50–59	1687 (50)	1260 (57)
	60–70	1691 (50)	966 (43)
	Total	3378 (100)	2226 (100)
			
NPI group	Excellent	186 (5)	423 (19)
	Good	474 (14)	682 (31)
	Moderate 1	569 (17)	440 (20)
	Moderate 2	598 (18)	213 (9)
	Poor	418 (12)	94 (4)
	Unknown	1113 (34)	374 (17)
	Total	3378 (100)	2226 (100)

NPI=Nottingham Prognostic Index.

**Table 2:** Cox's regression analysis from the univariate models for the separate effects of each of NPI, age and mode of detection on survival, and the multivariate model with each factor adjusted for the two others

**Factor**	**Category**	**Deaths**	**Relative hazard (95% CI) univariate Cox's regression results**	**Relative hazard (95% CI) multivariate Cox's regression results**
Age (years)	50–59	339	1.00 (—)	1.00 (—)
	60–70	443	1.41 (1.17–1.70)	1.36 (1.13–1.64)
				
NPI	Excellent	18	1.00 (—)	1.00 (—)
	Good	53	1.65 (0.96–2.82)	1.59 (0.93–2.72)
	Moderate 1	73	2.54 (1.51–4.26)	2.36 (1.40–3.97)
	Moderate 2	141	6.38 (3.94–10.42)	5.65 (3.43–9.30)
	Poor	188	15.65 (9.64–25.40)	13.87 (8.46–22.73)
				
Detection mode	Symptomatic	641	1.00 (—)	1.00 (—)
	Screen detected	141	0.43 (0.34–0.53)	0.79 (0.63–0.99)

CI=confidence interval; NPI=Nottingham Prognostic Index.

**Table 3:** Attenuation of the effect of screen detection on survival, after adjustment for different factors

**Factors**	**Relative hazard (95% CI), screen detected *vs* symptomatic**	**% of screen detection effect**
None	0.43 (0.34–0.53)	0
Size and node status	0.66 (0.53–0.82)	49
NPI	0.76 (0.60–0.95)	67
NPI, age (years)	0.79 (0.63–0.99)	72

CI=confidence interval; NPI=Nottingham Prognostic Index.

**Table 4:** Five-year overall survival (%)

**NPI**	**All patients**	**Symptomatic**	**Screen detected**
<2.4	96	94	98
2.4–3.39	93	93	94
3.4–4.39	90	89	93
4.4–5.39	79	78	88
5.4+	58	58	65

NPI=Nottingham Prognostic Index.
